# The Effectiveness of Narrative Versus Didactic Information Formats on Pregnant Women’s Knowledge, Risk Perception, Self-Efficacy, and Information Seeking Related to Climate Change Health Risks

**DOI:** 10.3390/ijerph17196969

**Published:** 2020-09-23

**Authors:** Adebanke L. Adebayo, Rochelle Davidson Mhonde, Nathaniel DeNicola, Edward Maibach

**Affiliations:** 1 Department at George Mason University, George Mason University, Fairfax, VA 22030, USA; rmhonde@gmu.edu; 2 School of Medicine and Health Sciences, The George Washington University, Washington, DC 20052, USA; ndenicola@gmail.com; 3 Center for Climate Change Communication, George Mason University, Fairfax, VA 22030, USA; emaibach@gmu.edu

**Keywords:** climate change, pregnant women, narrative communication, risk perception, information-seeking, self-efficacy

## Abstract

Climate change is a global threat that poses significant risks to pregnant women and to their developing fetus and newborn. Educating pregnant women about the risks to their pregnancy may improve maternal and child health outcomes. Prior research suggests that presenting health information in narrative format can be more effective than a didactic format. Hence, the purpose of this study was to test the effectiveness of two brief educational interventions in a diverse group of pregnant women (*n* = 151). Specifically, using a post-test only randomized experiment, we compared the effectiveness of brief information presented in a narrative format versus a didactic format; both information formats were also compared to a no information control group. Outcome measures included pregnant women’s actual and perceived knowledge, risk perception, affective assessment, self-efficacy, intention to take protective behaviors, and subsequent information seeking behavior. As hypothesized, for all outcome measures, the narrative format was more effective than the didactic format. These results suggest the benefits of a narrative approach (versus a didactic approach) to educating pregnant women about the maternal and child health threats posed by climate change. This study adds to a growing literature on the effectiveness of narrative-based approaches to health communication.

## 1. Introduction

Climate change is arguably the biggest global health threat of the 21st Century [1,2]. Climate change poses significant risks to the reproductive health of women across the lifespan as well as to developing fetuses and newborns [3,4]. Pregnant women, developing fetuses, and young children are considered to be one of the populations most at-risk of the adverse effects of climate change health risks, especially women in low-income and urban areas [5,6,7].

The U.S. National Climate and Health Assessment identifies eight key impacts of climate change on health, including health harms associated with heat waves, poor outdoor air quality (from smog and wildfires), extreme weather events, food- and water-related infections, vector-borne diseases, nutritional deficiencies, and mental health impacts [8]. Pregnant women and their fetuses are among the population most at-risk of these health harms, especially those resulting from extreme temperatures, poor air quality, food related infections, as well as mental health impacts. Exposure to air pollution and extreme heat both create elevated risk of serious obstetrical outcomes including preterm birth, low birthweight, and stillbirth [9,10,11]. Moreover, climate change is creating unprecedented exposures to extreme temperatures, smoke from wildfire, and other forms of air pollution in an expanding range of geographic locations [8].

During pregnancy, women’s ability to thermoregulate is compromised, making them particularly susceptible to heat waves [7,9]. In addition, prenatal and early childhood exposure to air pollution, especially PM2.5, contributes to respiratory illness as well as neurological damages [12]. The dangers of these environmental exposures have led the American College of Obstetricians and Gynecologists (ACOG) and the International Federation of Gynecology and Obstetrics (FIGO) to alert members of the reproductive and maternal health community about the far-reaching effects of climate change on maternal and neonatal health outcomes [10,13,14,15].

While the harmful impacts of climate change and air pollution on pregnant women and their fetuses are well-established [7,9,10,11,12], little research has been conducted to assess pregnant women’s understanding of their and their fetuses’ health risks resulting from climate change. Moreover, to the best of our knowledge, no educational interventions have been tested.

Flocks and colleagues conducted a qualitative study of female farmworker’s perceptions of heat related illness and pregnancy health [16]. The women understood that heat exposure can adversely affect pregnancy and fetal health, but they did not know which protective behaviors can mitigate against these risks. In a survey of the general public, Maibach and colleagues found that few Americans are able to name any specific health risks from climate change, or any groups of Americans who are particularly at-risk to the health risks associated with climate change [17]. Similarly, a more recent survey found that few Americans are able to name any specific health consequences from exposure to air pollution, or who is most likely to be harmed by such exposure [18].

Other studies of public understanding have shown that climate change knowledge is positively associated with climate change risk perceptions, and that perceived self-efficacy to respond to climate change is positively associated with both seeking information and engaging in other meaningful behavioral responses to climate change [19,20,21]. Moreover, other research has shown that the presentation of well-crafted education information can increase public understanding of climate change risks [21,22,23,24,25,26] which, in turn, can lead to meaningful behavioral responses. Therefore, using well-crafted narrative messages to educate pregnant women about their and their developing baby’s vulnerabilities to the harmful health effects of climate change, including the actions that can be taken to reduce the risks, may help motivate and enable women to take appropriate protective behaviors.

While people’s knowledge of their health risks from climate change is likely a necessary precondition for engaging in protective action, for most people it is not a sufficient precondition. Research across many domains of health behavior—and many other domains of behavior—has shown self-efficacy to be another necessary precondition for adoption and maintenance of self-protective action [27]. Self-efficacy refers to people’s confidence in their ability to perform a given action—self-protective or otherwise—under a range of conditions they would normally encounter. People with a strong sense of confidence in their ability to perform a given self-protective behavior are more likely to try the behavior and more likely to maintain the behavior over time. Therefore, educational materials about the health harms of climate change on pregnant women and their fetuses must not only increase women’s knowledge of their risks and protective actions, they must also increase women’s sense of self-efficacy in their ability to perform the self-protective actions.

A central challenge in all risk communication efforts—be they environmental health risks or otherwise—is how to gain and hold the attention of people at risk long enough to help them understand their risks and response options. Risk communication refers to the exchange of information, advice, and opinions between experts and people facing threats to their health, economic, or social well-being. The ultimate purpose of risk communication is to enable people at risk to make informed decisions to protect themselves and their loved ones [28]. Risk communication that fails to gain and hold people’s attention, however, is unlikely to achieve this aim [29].

There has been a considerable amount of research on the attributes of brief but effective presentation of risk information in such settings [30,31,32,33]. Risk communication is most likely to be successful when risk information is provided during the times and places that at-risk people are most likely to be receptive to the information [30]. For a variety of reasons, waiting rooms in health care facilities are often seen as an appropriate place to initiate risk communication for relevant health risks: the patients may be at risk; the context is congruent; they have the time and possibly the necessary focus to process risk information; any questions raised by information presented can be asked and answered in the medical visit that will soon be occurring [31,32].

Moreover, effective risk communication often reveals to people the need for additional information to find answers and reduce anxiety [27,29]. Therefore, subsequent information seeking is often considered an indicator of effective risk communication [29,34].

The research question that motivated our research was as follows: What form should brief information take to help pregnant women understand the health risks of climate change to pregnant women and their developing fetuses? Didactic information, which follows a largely scientific—or information deficit model—educational style, is the informational format traditionally used for providing factual knowledge to increase patient and public understanding of health risks and persuade audiences to behavioral change [35]. However, a rapidly growing literature suggests that health risk information delivered in a narrative form, which follows a story-driven format, may be more effective than similar information delivered in a traditional didactic form [36,37].

Stories typically focus on people—real or fictitious—although some stories feature nonhuman characters. Kreuter et al. define narrative “as a representation of connected events and characters that have an identifiable structure, is bounded in space and time, and contains implicit or explicit messages about the topic being addressed’’ [38] (p. 222). Niles asserts that because narrative is a universal form of human communication, humans would be aptly named *homo narrans*—storytellers; we organize information, create meaning, and shape our world through the social function of storytelling [39].

In a comprehensive review of the usefulness of narratives in health communication, Shaffer and colleagues make the case that the effectiveness of narratives stems both from its superior ability to communicate information, and to persuade—by changing people’s attitudes, judgement, and behaviors [40]. Specifically, narrative information more effectively attracts people’s attention than didactic information, is more easily processed, and is more easily and accurately retrieved. Information presented narratively is also less likely to elicit counter-arguing—thereby increasing persuasiveness of the information, which promotes attitude and behavior change.

To explain both the effectiveness of narratives—as well as their differential effectiveness (i.e., why some narratives are more effective than others)—Shaffer and colleagues posit three broad factors that determine the magnitude of narrative impact: audience interest (which includes source credibility as well as realism and entertainment value of the content); involvement (empathy, perspective-taking, and identification with the characters in the narrative); immersion (transportation of oneself into the story, engagement with the story, and simulation or the opportunity to practice how one would behave in the situation depicted) [40].

Narratives are particularly compelling when people become transported into or deeply immersed in the story [41]. Van Laer and colleagues conducted a meta-analysis of narrative research to identify the antecedents and consequences of narrative transportation [42]. Stories that have identifiable characters, an imaginable plot, and verisimilitude (i.e., they are realistic in the sense that they could happen) are more likely to be deeply immersive. Moreover, story recipients who are familiar with the topic of the story are more likely to become immersed.

Importantly, narrative information tends to engage audience members emotionally as well as cognitively, emotion being a well-established influence in persuasion [43]. Moreover, the emotional flow of a narrative, defined as “evolution of the emotional experience during exposure to the media message”, can play an important role in narrative transportation by heightening involvement in the storyline [44,45].

While many authors have commented on the imperative to use stories to heighten public understanding of and engagement in climate change [46,47], relatively little empirical research has been done to test the effectiveness of the narrative approach in this context [33,48,49]. Jones tested the impact of three brief narrative essays structured to align with different worldviews drawn from Cultural Theory, as compared to a list of scientific facts about climate change. Counter to his hypotheses, the narrative essays were not more transportive than the list of factual statements, but this may have been due to the fact that the narrative essays were more factually based than story based—invoking organizations rather than people [48]. Conversely, in a qualitative examination of the impact of a documentary television series about climate change that was strongly narratively driven (The Years of Living Dangerously), Bieniek-Tobasco and colleagues (2019) found that watching the content increased viewer’s concerns about climate change, but left them with an attenuated sense of efficacy in their ability to take meaningful actions to address the problem [49]. However, in another study, Bieniek-Tobasco and colleagues randomly assigned approximately 2000 people to watch one of several episodes of the TV series or a control video (about transmission of diseases from animals to humans); as hypothesized, the climate narratives significantly increased viewer’s risk perceptions and their sense of efficacy to respond to the risk [50]. Other studies have shown that narrative messages increase interest, learning, and behavioral intentions for a range of health issues [51,52].

In this study, we compared the effectiveness of brief narrative-based information about the maternal and child health risks associated with climate change to comparable information presented in a didactic format. The indicators of effectiveness in our study were knowledge of maternal and child health risks associated with climate change, perceived risk to self and pregnant women in general, affective assessment of climate change, self-efficacy to reduce the maternal and child risks of climate change, intention to engage in risk-reducing actions, and subsequent information seeking behavior. Specifically, we assessed information seeking behavior by offering research participants the opportunity to download a free app provided by the Environmental Protection Agency (EPA) called AirNow. AirNow provides daily air quality forecasts for local, national, and global locations and is supported by the EPA, Center for Disease Control, National Aeronautics and Space Adminstration (NASA), and other credible national organizations. AirNow is designed with an interactive color-coded index to alert people when the outdoor air quality is at unhealthy levels in their city, so that they can avoid exposure to the extent possible [53]. By downloading AirNow, pregnant women in the United States (and others) can proactively engage in information seeking that offers a directly actionable means of reducing one form of health risks associated with climate change—prolonged exposure to unhealthful outdoor air.

We tested the following hypotheses:

**Hypothesis** **(1).** *Narrative information will be more effective than traditional information at enhancing pregnant women’s understanding of and response to the maternal and child health risks of climate change. Specifically, narrative messages will be superior at enhancing*:

**Hypothesis** **(1a).** *Perceived knowledge of the health risks associated with climate change.*

**Hypothesis** **(1b).** *Actual knowledge of the health risks associated with climate change.*

**Hypothesis** **(1c).** *Perceived risk of maternal and child health harms from climate change.*

**Hypothesis** **(1d).** *Affective assessment of climate change.*

**Hypothesis** **(1e).** *Self-efficacy in reducing the health risks associated with climate change.*

**Hypothesis** **(1f).** *Intention to engage in health risk reducing behavior related to climate change.*

**Hypothesis** **(1g).** *Subsequent information-seeking behavior.*

## 2. Materials and Methods

### 2.1. IRB Approval

This research was approved by George Mason University Institutional Review Board: approval #1404857.

### 2.2. Experimental Design

We conducted a randomized experiment with two treatment groups and one control group, conducting post-test measurements only. We ruled out the possibility of conducting pretest measures due to a concern about sensitizing study participants; the downside of this design decision is that we were not able to control for participant’s pre-existing views.

### 2.3. Research Recruitment

All research participants were pregnant women recruited through convenience sampling in the waiting rooms of a diverse set of private obstetrical practices (*n* = 2) and hospital clinics (*n* = 2) in Washington, DC, Largo, Maryland, and Maryville, Illinois. One of the hospital-based practices specifically serves women with high-risk pregnancies. These locations were selected to ensure a diverse group of participants in terms of race/ethnicity, socio-economic status, and geography, and because the investigators had access to them. As the research materials were in English, only English-speaking patients were asked to participate.

Participants were approached in the waiting room by one of the study authors (A.L.A. or R.D.M.) and asked if they would agree to participate in a research study about maternal and child health risks. Women willing to participate were given an electronic mobile tablet (similar to an iPad) that was outfitted with Qualtrics survey software. The Qualtrics survey software randomly assigned participants to a treatment condition (narrative information, didactic information) or to a no information control group, administered the consent form, presented the treatment material (in the case of the two informational conditions), and then posed a series of closed-ended questions to assess the outcome measures and demographic questions (age, race, gestational age, holder of insurance, and state of residence). On the final survey screen, participants were introduced to the EPA AirNow app, and asked if they would like to be taken to the EPA website to learn how they can download the app to their phone. The survey was designed to be completed in 5–7 min; on average, it took participants 5 min to complete the study.

### 2.4. Treatments

The didactic information treatment was an abbreviated version of the Environmental Protection Agency’s brochure titled *“Climate Change and the Health of Pregnant Women”* [54]. After reviewing the brochure to identify the important factual information conveyed, we edited out additional information that we deemed less important or confusing. A copy of the edited brochure is included in Supplementary Materials.

We then used the information in the modified EPA brochure to provide the factual content for the narrative presentation—which took the form of a full-color comic book (see Supplementary Materials). This was an important design feature in our research because it ensured consistency of the factual information provided in both treatment conditions. The characters in the comic book were a pregnant woman (the protagonist), her doctor, her child, and one of her pregnant friends. The story presents her interactions with her doctor, her child, and her friend. During these interactions, she learns about the maternal and child health risks of climate change from her doctor and shares the information with her child and friend. The protagonist also suggested to her child that they avoid the risky behavior, thereby in effect modeling the recommended behavior, which should increase the reader’s sense of self-efficacy.

### 2.5. Measures

With two exceptions, all outcome measure items (detailed below) were measured on a 5-point Likert scale (1 = strongly disagree; 2 = disagree; 3 = neutral; 4 = agree; 5 = strongly agree). For outcome measures in which more than one question was asked, the answers to each question were combined in a simple additive scale.

With the one exception noted below, all measures were developed by the investigators specifically for this study. All of the measures were pilot tested before they were used in this study. The full instrument is presented in Appendix A.

Perceived knowledge: Perceived knowledge was measured with three items that assessed participants’ perceptions of their knowledge about the health relevance of climate change including: “I know the ways that global warming could hurt me or my developing baby”.

Actual knowledge: Actual knowledge was assessed with four questions about climate change and how it can adversely affect maternal and child health, including: “Global warming is causing longer and hotter heat waves”. The Likert-type response scale was used to give respondents the ability to express the certainty of their knowledge (by agreeing or disagreeing either somewhat or strongly).

Perceived risk: Perceived risk was assessed with three questions that examined perceptions of climate change risk as it affects pregnant women and their developing babies. For example: “I believe global warming can hurt me and my developing baby”;

Affective Assessment: Affective assessment of global warming was measured with a single question that examined participant’s perceived risk of climate change as a feeling: “Do you think global warming is a good or bad thing?” The 6-point response scale ranged from very bad (−3) to very good (+3). This measure has been used in prior research [55].

Self-efficacy: Self-efficacy was assessed with two questions that measured participant’s confidence in their ability to take actions to protect against specific impacts of climate change on their health during their pregnancy. For example: “I am confident I can protect myself and my developing baby from heat waves”.

Behavioral intention: Behavioral intention was assessed with four questions that assess participant’s intention to perform specific actions that pregnant women can take to protect themselves from the adverse effect on climate change. For example: “I plan to download a free air quality app on my phone so I can check the air quality”.

Information seeking behavior: Information seeking behavior was measured as a dichotomous measure based on whether or not the participant chose to go to the EPA website to learn more about the AirNow app (coded 1 = yes or 0 = no). At the conclusion of the survey, participants who selected “yes” were automatically redirected to the webpage where the AirNow app could be downloaded.

### 2.6. Participants

A total of 151 pregnant women participated in the study. Their mean age was 28.23 (SD = 5.89). Their mean gestational age was 25.91 weeks (SD = 10.11), with a minimum gestational age of 4 weeks and a maximum of 40 weeks. A majority of the participants were Black/African American (54.3%, *n* = 82); the others were White (23.2%, *n* = 35), Hispanic/Latino (6.6%, *n* = 10), Asian (6.6%, *n* = 10), “other” (3.3%, *n* = 5), and no response to the race/ethnicity question (6%, *n* = 9). All of the participants indicated that they have health insurance, with a majority having Medicaid (58.3%; *n* = 88), others having private insurance (41.7%; *n* = 63),

## 3. Results

To test the hypotheses, we conducted a Multivariate Analysis of Variance (MANOVA) to assess between-group differences (narrative, didactic, and no information control) on measures of perceived knowledge, actual knowledge, risk perception, affective assessment, self-efficacy, and behavioral intentions. As a prior step, to determine the appropriateness of MANOVA, we assessed the correlations between all dependent variables in order (see Table 1); Box’s M test for the equality of covariance matrices was not significant, *F*(30, 61771.03) = 41.84, *p* < 0.111, indicating that the data has homogeneity of variance and covariances.

The omnibus MANOVA showed a statistically significant difference between the three conditions, Wilks’ Λ = 0.73, *F*(10, 274) = 4.45, *p* < 0.001, partial ηp2 = 0.14, power = 0.99. The univariate ANOVA showed that there were significant between-group differences for each of the outcome measures: perceived knowledge *F*(2, 141) = 14.4, *p* < 0.001, η2 = 0.17, power = 0.99; actual knowledge *F*(2, 141) = 5.71, *p* = 0.004, η2 = 0.07, power = 0.86; perceived risk *F*(2, 141) = 6.95, *p* = 0.001, η2 = 0.09, power = 0.92; affective assessment *F*(2, 141) = 6.38, *p* < 0.002, η2 = 0.09, power = 0.89; self-efficacy *F*(2, 141) = 15.21, *p* < 0.001, η2 = 0.18, power = 0.99; and behavioral intention *F*(2, 141) = 9.62, *p* < 0.001, η2 = 0.12, power = 0.98.

Post hoc testing was conducted to identify which conditions were significantly different. Participants in the narrative condition had significantly higher measures of perceived knowledge than those in the didactic condition *p* = 0.001, and the no treatment conditions *p* < 0.001. Thus, H1a was supported. Participants in the narrative condition had a significantly higher actual knowledge than those in the no treatment condition *p* = 0.003, although they were not significantly more knowledgeable than those in the didactic condition. Thus, H1b was partially supported. Similarly, participants in the narrative condition had significantly higher perceived risk than those the didactic *p* = 0.04, and the no treatment conditions, *p* = 0.001. Thus, H1c was supported. Participants in the narrative condition had significantly lower affective assessments of global warming than those in the didactic condition, *p* = 0.019, and the no treatment condition, *p* < 0.008. Thus, H1d was supported. Participants in the narrative condition had significantly higher perceived self-efficacy than participants in the didactic, *p* = 0.01, and the no treatment conditions, *p* < 0.001. Thus, H1e was supported. Participants in the narrative condition had significantly higher behavioral intentions than those in the didactic, *p* = 0.019, and the no treatment conditions, *p* < 0.001. Thus, H1f was supported. See Table 2 for means and standard deviations.

To test Hypothesis 1g, we conducted a logistic regression to ascertain which information condition was most strongly associated with going to the EPA website to download the AirNow app. The logistic regression was statistically significant at χ2(1) = 17.402, *p* < 0.001. The model explained 24% (Nagelkerke R2) of the variance in information seeking. The model was able to correctly classify 84% of those who requested to be taken to the EPA AirNow site and 60% of those who did not, for an overall success rate of 69%. When compared to the didactic condition, participants in the narrative condition were 7.98 times more likely to engage in this form of information seeking (see Table 3). Thus, H1g was supported, and overall, H1 was fully supported.

## 4. Discussion

This study examined the effectiveness of traditional didactic information versus narrative information on pregnant women’s perceptions of climate change. While prior research has shown that didactic information about climate change can be effective [56], our study found that narratively based information was more effective than didactic information in increasing pregnant women’s knowledge, risk perceptions, self-efficacy, and intentions to adopt risk-reducing behavior, and far more effective at influencing their subsequent actual information seeking behavior—even though the factual content of the two forms of information were equivalent.

This finding is consistent with the small but developing literature which points to the persuasive superiority of narratively based information versus didactically based information, at least with some people, in some settings [35,36,51,52,57]. The attention-getting quality of narratively based information, and its ability to heighten reader’s engagement with content, make it particularly well suited educating patients in waiting rooms, which is an educational setting that has some obvious inherent limitations (e.g., physical discomfort, social discomfort, competing sources of auditory and visual information—often from a television, etc.). In such attention- and time-limited environments, the advantages of narratively driven information to earn people’s attention and persuasively deliver risk-reducing information can potentially have important subsequent health and environmental benefits.

The brevity of the educational interventions is worth noting. On average, women took only 5 min to participate in this study, including the time necessary to read the educational information (in the case of women in the two treatment conditions) and answer all of the assessment questions. Therefore, in environments with attention and time constraints, it is important to use engaging materials to maximize both time and attention to the message; hence narrative communications would be preferable to didactic materials in such contexts. We believe this heightens the generalizability of our findings, given the prevalence of short-format patient education materials in health care settings.

With one exception (discussed below), the difference in outcomes between the narrative and the didactic information conditions were of a medium effect size (with Cohen’s d-scores ranging from 0.35 to 0.75). To put these differences in context, it is helpful to keep in mind that a one point shift upward on most of our scales represents a move from a neutral response to agreement with the statement (for example, “I believe that global warming is harmful for pregnant women”), or from agreement to strong agreement.

The one dependent measure that showed a larger than medium effect size was the information seeking behavior measure: choosing to go to the EPA website to learn more about the AirNow app, a localized air quality monitoring tool that can help people limit their exposure to unhealthful outdoor air pollution. The difference between the narrative and didactic information conditions was very large: women who read the narrative information were nearly eight times more likely to go to the EPA AirNow website than women in the didactic information condition. The fact that narratively delivered information had a bigger impact on participant’s information seeking behavior than on their knowledge, risk perceptions, and self-efficacy is an intriguing and potentially important finding worthy of further research.

One surprising finding from this study is that pregnant women who read the information presented in a didactic format were substantially less likely to seek additional information—about how to download the EPA AirNow app—than pregnant women who received no information at all (i.e., women in the control condition). In other words, the traditional didactic information format generated the opposite effect of what was intended, which is known as a “boomerang effect”. According to Hart, there are two distinct sets of causes of boomerang effects [58]. The first set of causes happen when people understand the information as intended, but their response to the information makes them less likely to perform the behavior than they otherwise would have. Examples include psychological reactance (when the recommendation makes people angry and they respond by behaving in the opposite manner) and fear control (when people feel incapable of performing the recommended action and respond by dismissing the validity of the threat) [59,60]. The second set of causes happen when people either do not understand the information as intended, or when additional information that is presented unintentionally counteracts the recommendation. Examples include accessibility or priming (when an undesirable set of mental associations are activated by exposure to the intended information) and peripheral processing (when people who lack motivation to pay careful attention to the information instead pay attention to peripheral elements of the presentation—which sometimes leads them to erroneous conclusions). Further research is required to determine which of these mechanisms may be responsible for the boomerang effect found in this study.

Like all research, this study is subject to a number of limitations. Our sample was a nonrepresentative convenience sample; thus, our findings cannot be considered representative of all pregnant women in the United States. That said, our sample was more ethnically diverse than the American population at large, and more likely to have Medicaid rather than private insurance—therefore they were likely to be at higher than average risk for adverse pregnancy outcomes. Thus, the strength of our findings might be attenuated in a lower-risk population.

It is also important to note that participation took place in the waiting rooms of obstetrical practices or hospital clinics where some of the participants were experiencing time constraints and anxiety that may have limited their attention span. Replication research conducted in different educational settings (e.g., online), and with other samples of pregnant women, is warranted.

Another limitation of our research is that, because we did not assess participant’s literacy skills, we were not able to assess the differential effectiveness of narrative and traditional information specifically with pregnant women with varying degrees of comprehension and literacy. Nevertheless, prior research has found narrative forms of communication to be especially effective with people who have low-literacy skills [56]. Low-literate pregnant women are likely to be at even higher levels of risk from climate change related health harms than high-literate pregnant women, because people with less household income tend to be more at risk for a range of health harms from climate change than people from higher income households [8]. Therefore, it is likely our narrative education might be even more effective among low-literate pregnant women.

A final limitation is the lack of longitudinal follow-up of the research participants to determine if the educational outcomes resulted in uptake of risk mitigation actions throughout their pregnancy. Longitudinal research of this type is warranted.

In the last several years, research around the effect of climate change to maternal and fetal health has increased, nevertheless gaps remain. This study presents important practical implications for future climate change communication and communication science research. Narrative information plays a key role in human communication. Our findings support utilizing this form of climate change education to foster self-protective behavior among pregnant women, a population that is at-risk to health harms from climate change. Narrative messages should be used to create educational interventions that mitigate the adverse effects of climate change on maternal and neonatal health outcomes.

It is important to note that educating pregnant women about their risks from climate change and related conditions is only one of many educational priorities that can improve birth outcomes—indeed it may be a low priority educational objective for pregnant women in locations with mild climates and with good air quality. The main finding of our research—that narratively based educational information can be more effective than traditional information-based education—may, however, have value for improving pregnancy education about other, potentially more important, pregnancy education topics.

## 5. Conclusions

The Intergovernmental Panel on Climate Change (IPCC) has identified pregnant women and their developing babies as a population that is particularly at-risk to the risks of climate change [5]. This study is the only study to date that tests educational methods intended to improve pregnant women’s understanding of their risk and give them risk management strategies. Our findings demonstrate the persuasive superiority of narrative information versus a traditional didactic information format on a range of important measures including knowledge, risk perception, self-efficacy and information seeking behavior. This educational intervention may have value beyond reducing climate change-related risks during pregnancy; narratively based educational information may be a better way to educate about a wide range of pregnancy-related risks.

## Figures and Tables

**Table 1:** Descriptive statistics and Pearson correlations of dependent variables.

Dependent Variable	α	M	SD	1	2	3	4	5	6
1 Perceived knowledge	0.83	3.60	1.03	-	0.69 *	0.57 *	−0.25 *	0.60 *	−0.63
2 Actual knowledge	0.94	4.03	1.02		-	0.80 *	−0.40 *	0.34 *	−0.67
3 Risk perception	0.86	3.82	1.03			-	−0.44 *	0.26 *	−0.61
4 Affective assessment	-	−1.62	1.79				-	−0.07	−0.31
5 Self-efficacy	0.89	3.56	0.98					-	−0.55
6 Behavioral intention	0.85	3.62	0.92						-

Note. All correlations are significant at * *p* < 0.01 (*p* is significant at 0.05), *n* = 151.

**Table 2:** Narrative, didactic, and control groups’ descriptive statistics.

Dependent Variables	Narrative	Didactic	Control
Perceived knowledge	4.16 (0.99)	3.44 (0.94)	3.17 (0.88)
Actual knowledge	4.37 (0.98)	4.04 (0.89)	3.69 (1.06)
Risk perception	4.23 (0.86)	3.74 (1.04)	3.49 (1.07)
Affective assessment	−2.29 (1.25)	−1.24 (1.97)	−1.21 (1.91)
Self-efficacy	4.06 (0.83)	3.54 (0.94)	3.06 (0.90)
Behavioral intention	4.03 (0.86)	3.54 (0.85)	3.27 (0.91)

Note. Means and standard deviation in parenthesis. Narrative condition (*N* = 52), Didactic (*N* = 49), Control (*N* = 50).

**Table 3:** Logistic regression for information seeking behavior.

Predictor	B	S.E.	Wald χ2	*df*	*p*	OR	95% CI
Control						reference	
Didactic vs. Cont.	−0.85	0.59	2.1	1	0.152	0.43	(134–1.37)
Narrative vs. Cont.	1.23	0.45	7.44	1	0.006	3.42	(1.41–8.25)
Narrative vs. Did.	2.07	0.56	13.92	1	0.001	7.98	(2.86–23.76)

Note: Information seeking measure was taken after random assignment to condition (control, traditional, narrative). OR = odds ratio; CI = confidence intervals.

## Supplementary Materials

The following are available online at https://www.mdpi.com/1660-4601/17/19/6969/s1.

## Appendix A

Measures

(* Reverse code 2, 8, 12)

Perceived Risk

1. I believe that global warming is harmful for pregnant women
2. I believe that global warming does not harm developing babies *
3. I believe global warming can hurt me and my developing baby

Perceived Knowledge

4. I know the ways that global warming could hurt me or my developing baby
5. I know how to protect myself and my developing baby from harm caused by heat waves
6. I know how to protect myself and my developing baby from harm caused by air pollution

Actual Knowledge

7. Global warming is causing longer and hotter heat waves
8. Global warming does not cause air pollution to get worse on hot days *
9. Air pollution can be harmful to pregnant women and their developing baby
10. Heat waves can be harmful to pregnant women and their developing babies

Self-Efficacy

11. I am confident I can protect myself and my developing baby from heat waves
12. I am not confident I can protect myself and my developing baby from air pollution *

Behavioral Intention

13. I plan to download a free air quality app on my phone so I can check the air quality
14. While I am pregnant, I plan to stay indoors as much as possible when the air quality is bad outside
15. While I am pregnant, I plan to stay indoors as much as possible when there is a heat wave
16. While I am pregnant, I plan to ask my doctor or nurse more information about how to protect myself and my baby from global warming
