# Supplementary material for: The Effectiveness of Narrative Versus Didactic Information Formats on Pregnant Women’s Knowledge, Risk Perception, Self-Efficacy, and Information Seeking Related to Climate Change Health Risks
Source: Int J Environ Res Public Health. 2020 Sep 23;17(19):6969. doi: 10.3390/ijerph17196969 (PMC7579394; doi:10.3390/ijerph17196969)
Supplement: Supplementary file 1 [file ijerph-17-06969-s001.pdf]

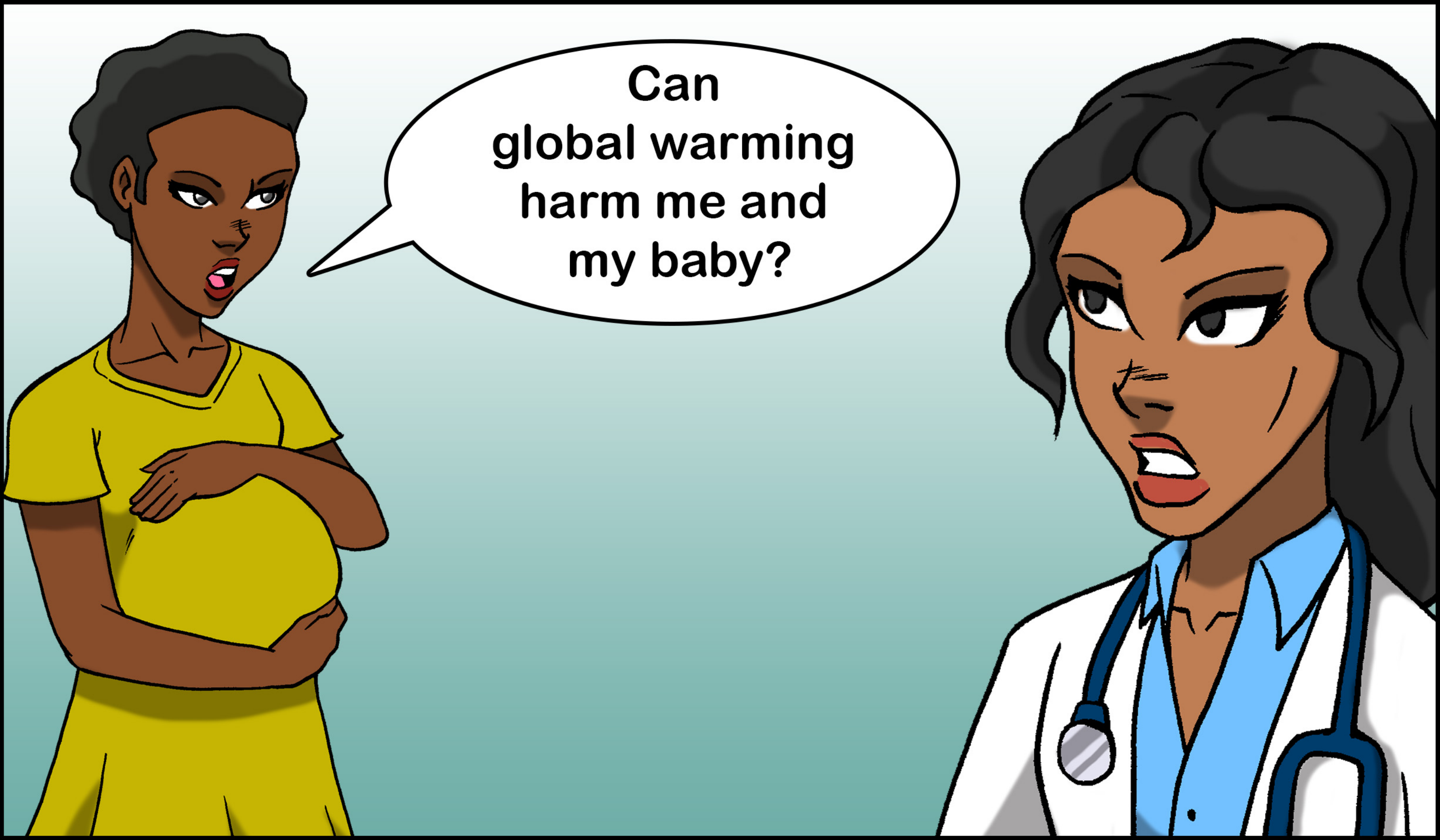
Can global warming harm me and my baby?

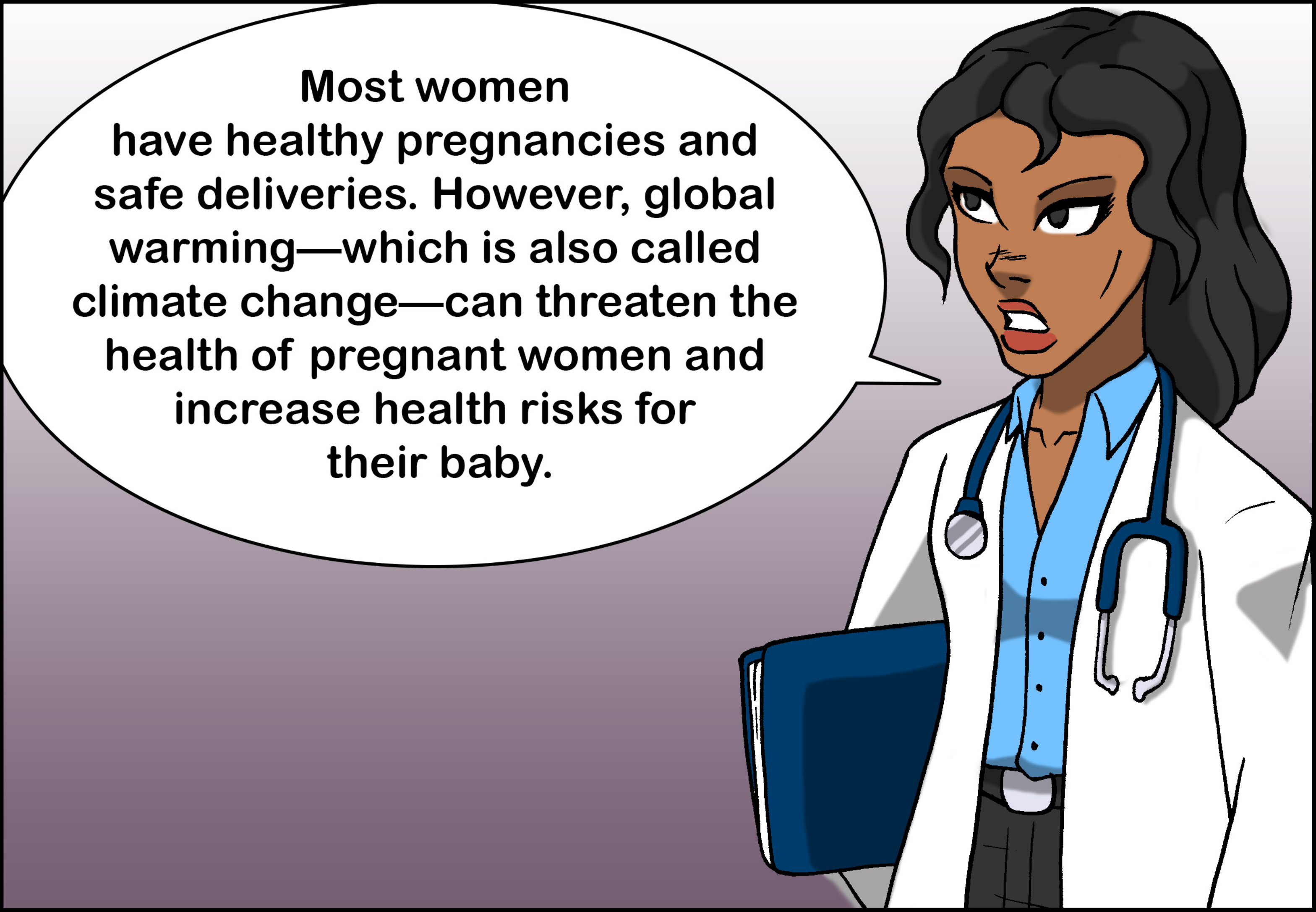
Most women have healthy pregnancies and safe deliveries. However, global warming—which is also called climate change—can threaten the health of pregnant women and increase health risks for their baby.

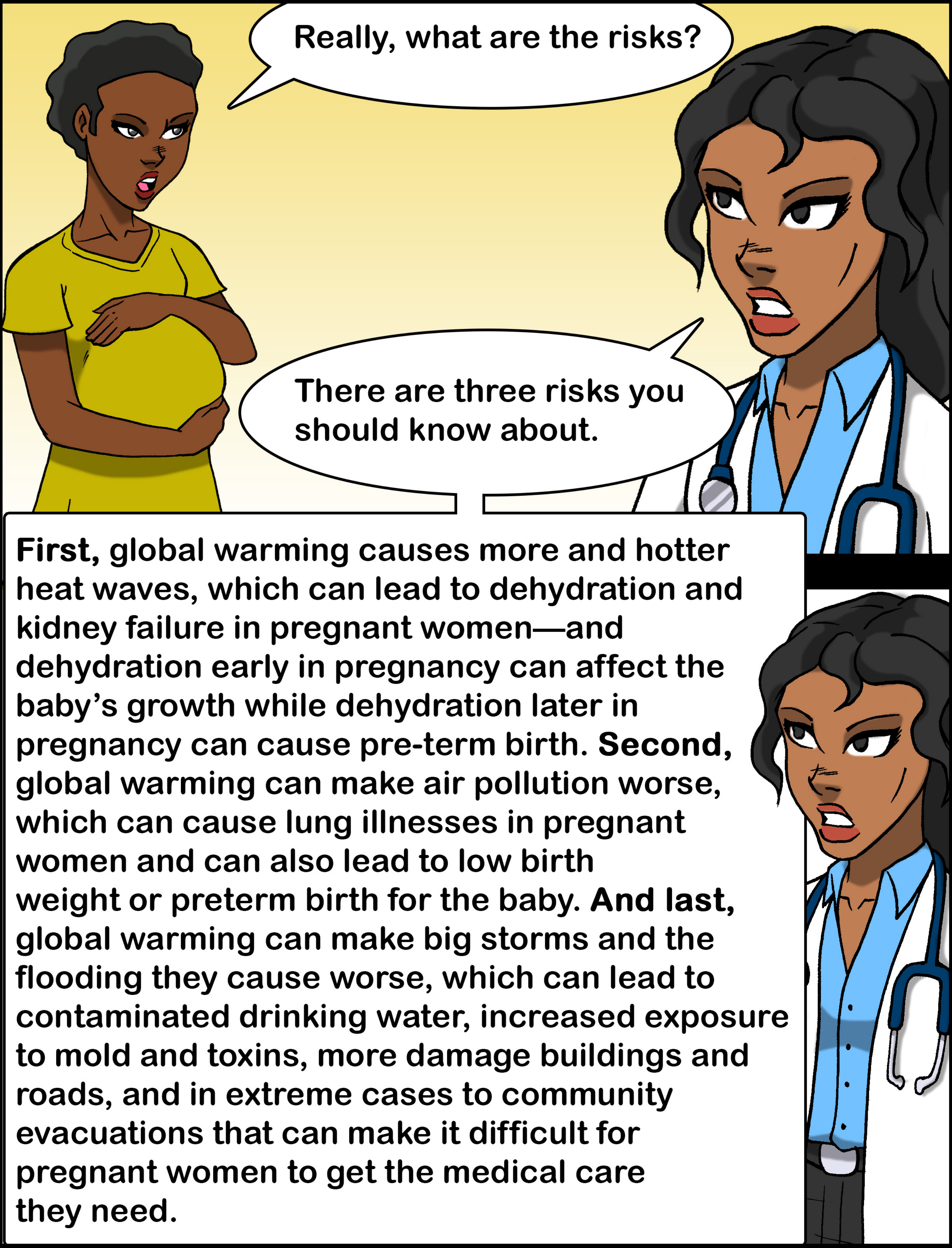
Really, what are the risks?
There are three risks you should know about.
**First,** global warming causes more and hotter heat waves, which can lead to dehydration and kidney failure in pregnant women—and dehydration early in pregnancy can affect the baby's growth while dehydration later in pregnancy can cause pre-term birth. **Second,** global warming can make air pollution worse, which can cause lung illnesses in pregnant women and can also lead to low birth weight or preterm birth for the baby. **And last,** global warming can make big storms and the flooding they cause worse, which can lead to contaminated drinking water, increased exposure to mold and toxins, more damage buildings and roads, and in extreme cases to community evacuations that can make it difficult for pregnant women to get the medical care they need.

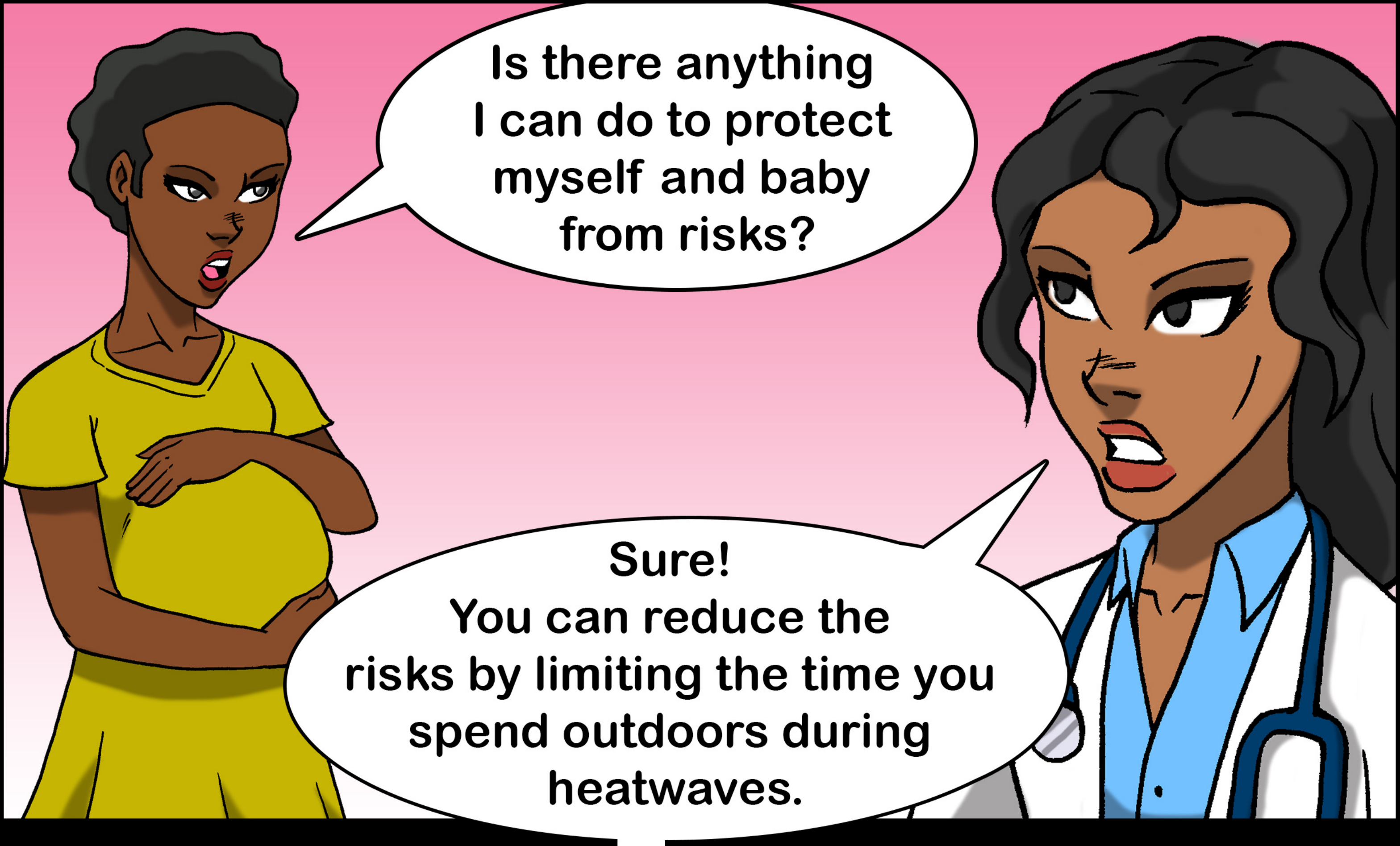
Is there anything I can do to protect myself and baby from risks?
Sure! You can reduce the risks by limiting the time you spend outdoors during heatwaves.

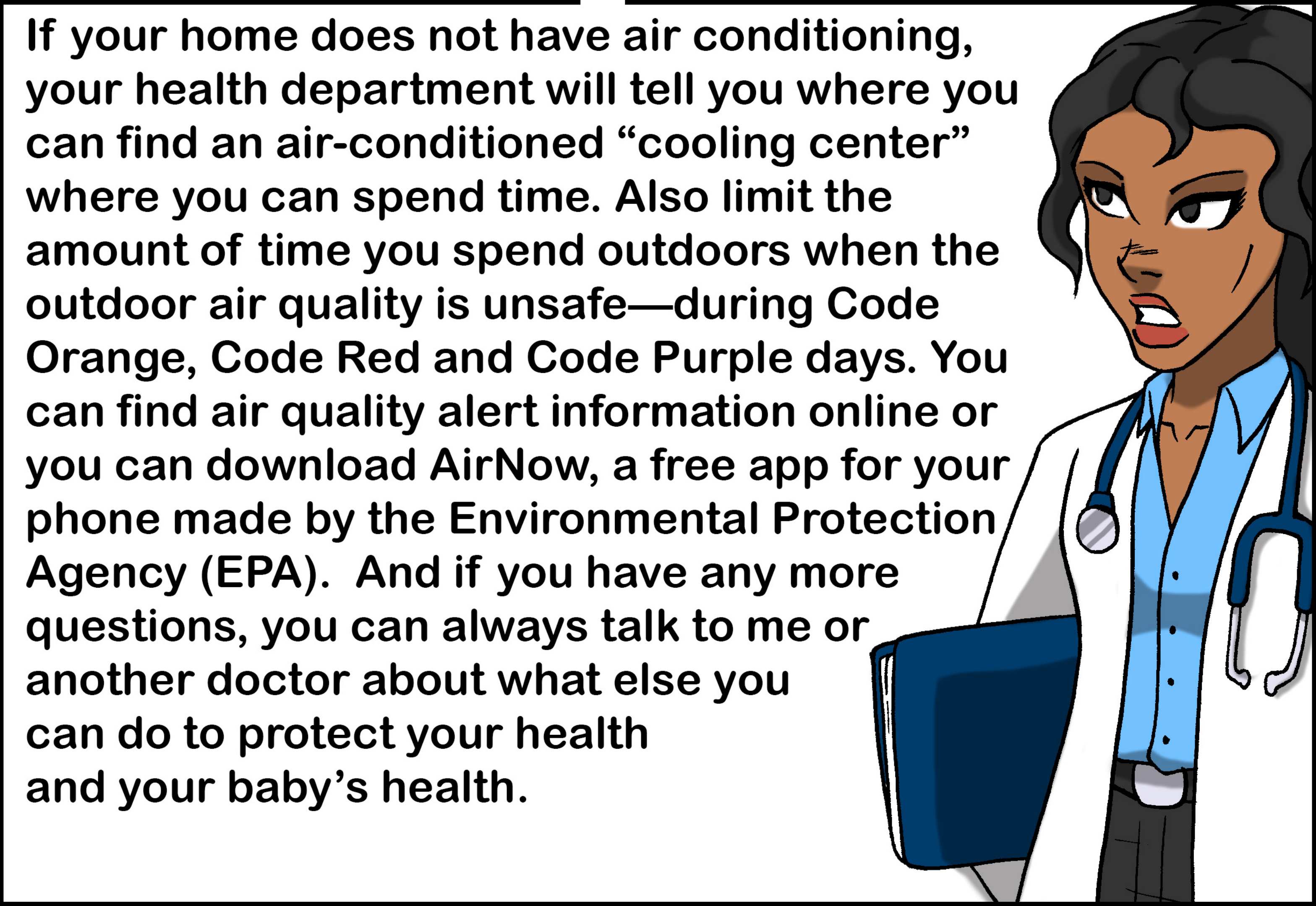
If your home does not have air conditioning, your health department will tell you where you can find an air-conditioned “cooling center” where you can spend time. Also limit the amount of time you spend outdoors when the outdoor air quality is unsafe—during Code Orange, Code Red and Code Purple days. You can find air quality alert information online or you can download AirNow, a free app for your phone made by the Environmental Protection Agency (EPA). And if you have any more questions, you can always talk to me or another doctor about what else you can do to protect your health and your baby’s health.

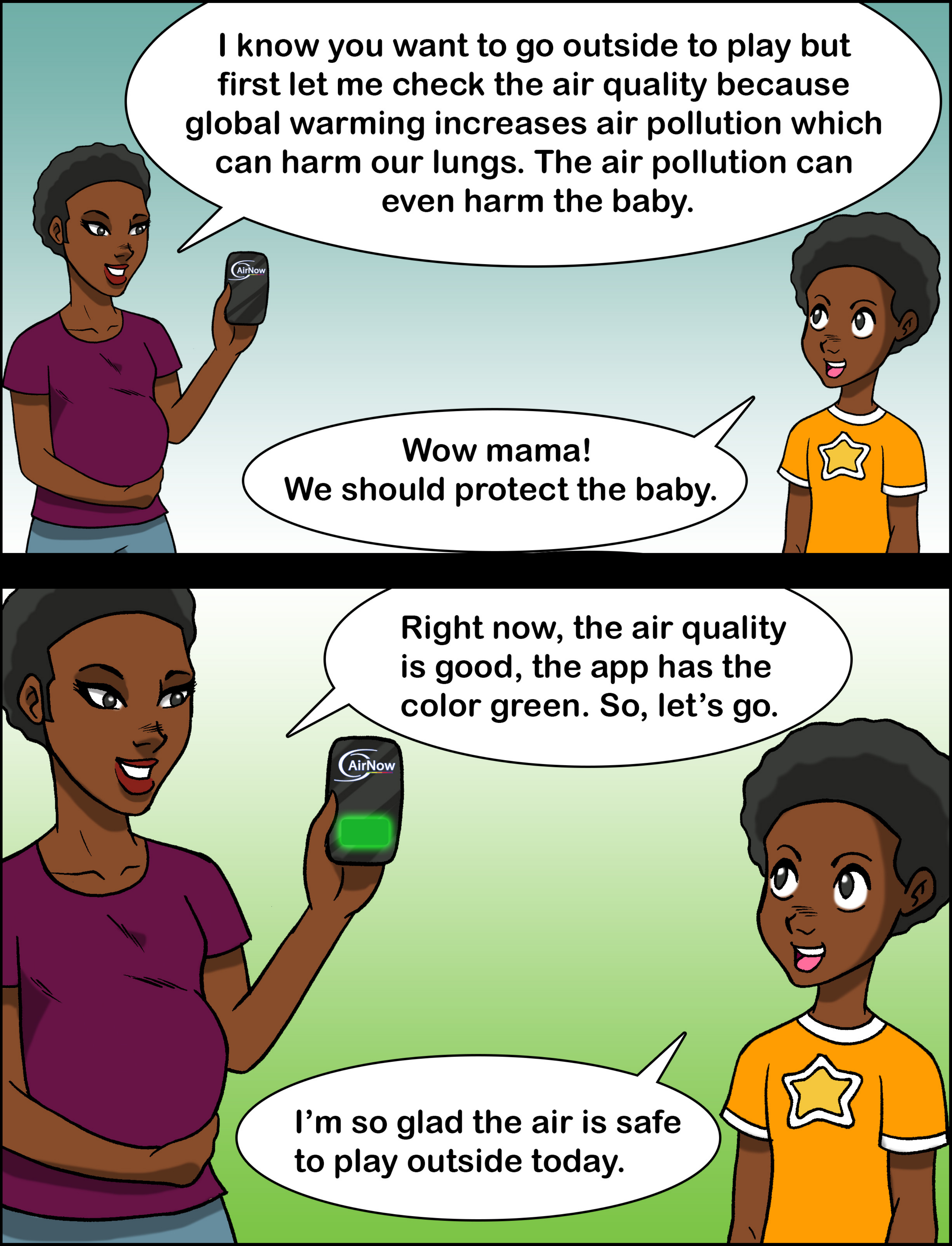
I know you want to go outside to play but first let me check the air quality because global warming increases air pollution which can harm our lungs. The air pollution can even harm the baby.
AirNow
Wow mama!
We should protect the baby.
Right now, the air quality is good, the app has the color green. So, let's go.
AirNow
I'm so glad the air is safe to play outside today.

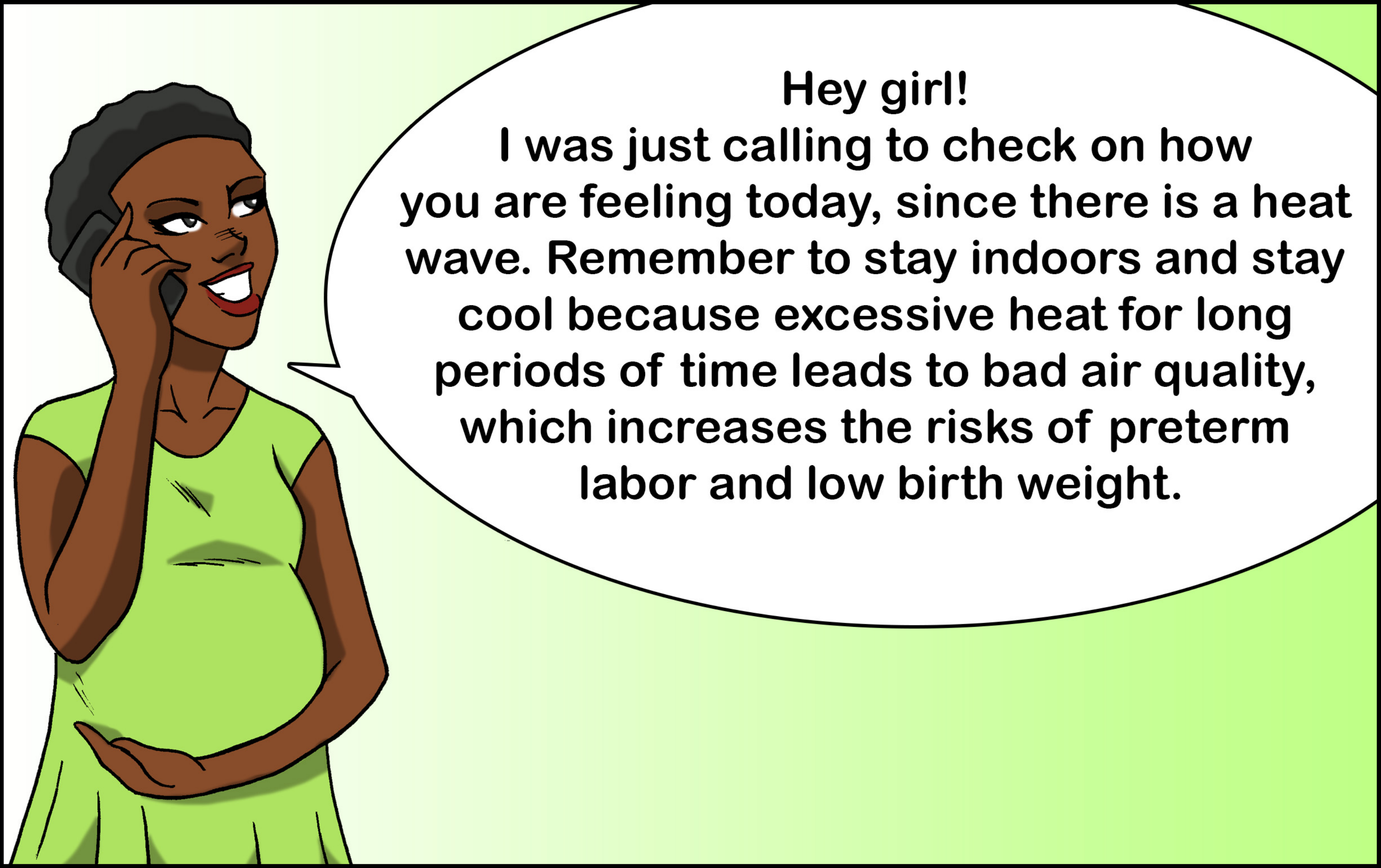
Hey girl! I was just calling to check on how you are feeling today, since there is a heat wave. Remember to stay indoors and stay cool because excessive heat for long periods of time leads to bad air quality, which increases the risks of preterm labor and low birth weight.

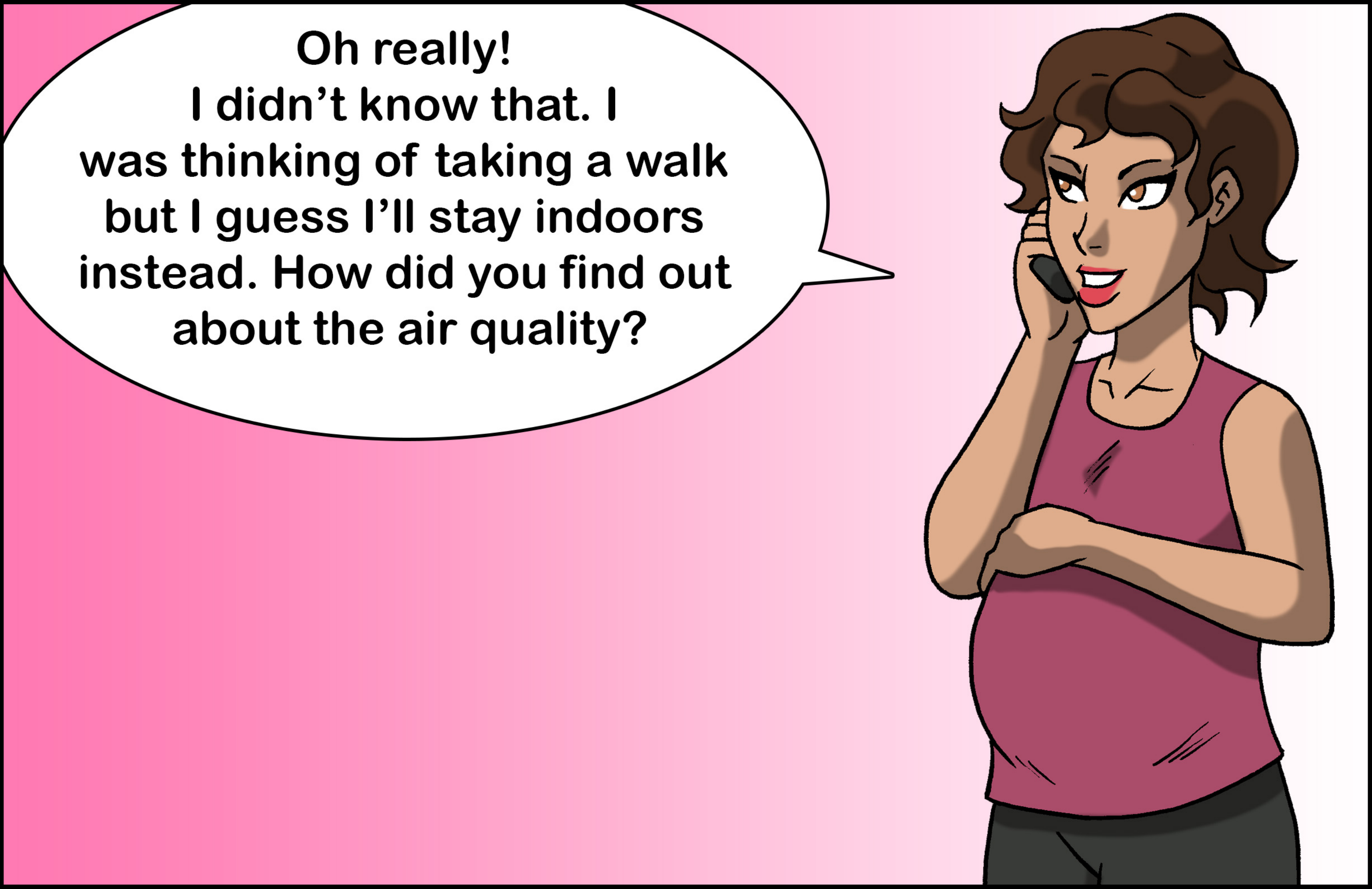
Oh really! I didn't know that. I was thinking of taking a walk but I guess I'll stay indoors instead. How did you find out about the air quality?

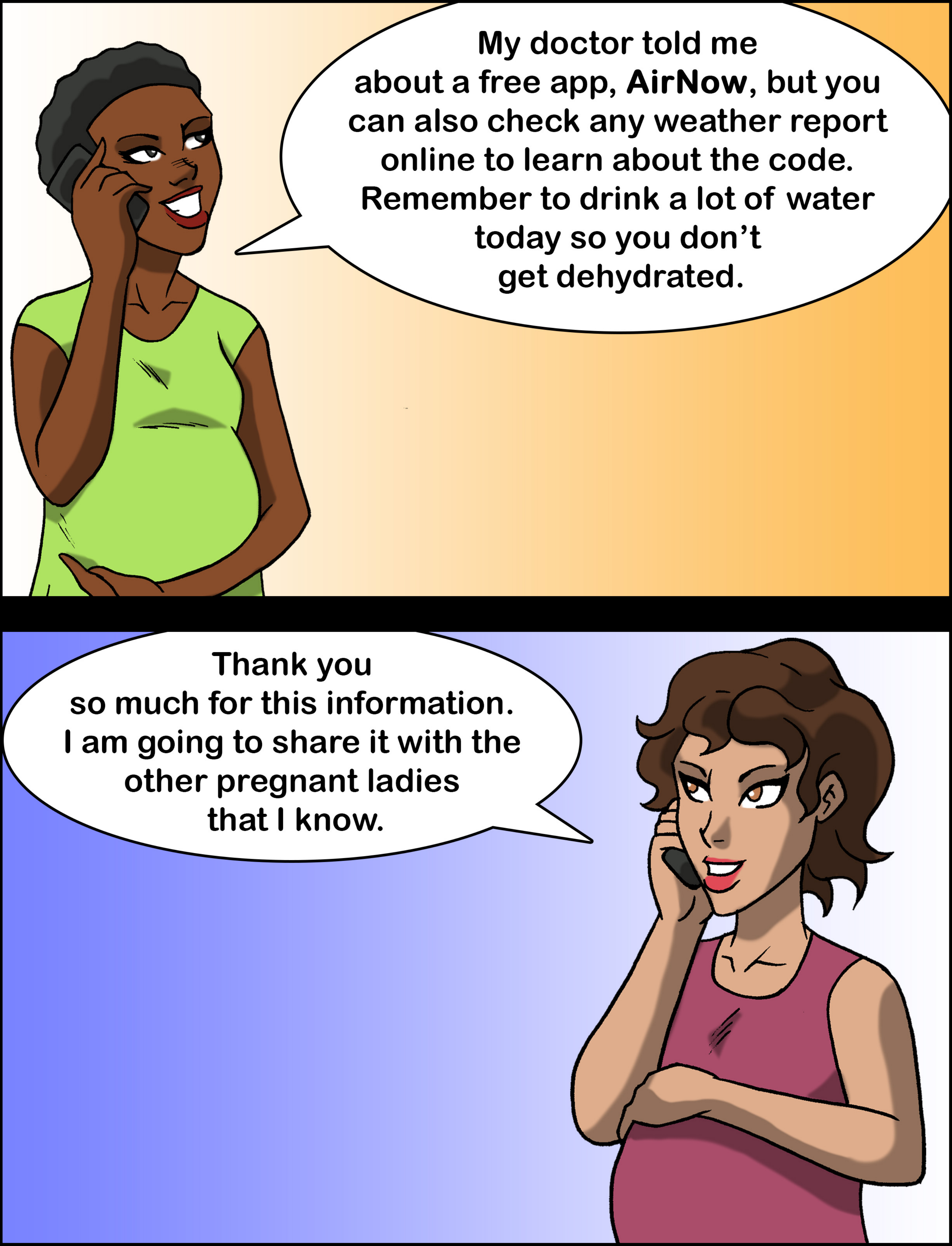
My doctor told me about a free app, AirNow, but you can also check any weather report online to learn about the code. Remember to drink a lot of water today so you don't get dehydrated.
Thank you so much for this information. I am going to share it with the other pregnant ladies that I know.

PLEASE READ THE FOLLOWING CAREFULLY BECAUSE AFTERWARD YOU WILL BE ASKED A FEW QUESTIONS ABOUT IT

Most women have healthy pregnancies and healthy babies. However, global warming—which is also called climate change—can threaten the health of pregnant women and increase health risks for their baby.

A factsheet from the U.S. Environmental Protection Agency (EPA)—Climate Change and the Health of Pregnant Women—explains that global warming can affect the health of pregnant women and babies in these ways:

- Global warming causes more and hotter heat waves, which can lead to dehydration and kidney failure in pregnant women. Dehydration early in pregnancy can affect the baby's growth while dehydration later in pregnancy can cause pre-term birth.
- Global warming can make air pollution worse, which can cause lung illnesses in pregnant women and can also lead to low birth weight or pre-term birth for the baby.
- Global warming can make big storms and the flooding they cause worse, which can lead to contaminated drinking water, increased exposure to mold and toxins, more damage buildings and roads, and in extreme cases to community evacuations that can make it difficult for pregnant women to get the medical care they need.

There are things you can do to reduce health risks to you and your baby:

- During heat waves, limit the amount of time you spend outdoors. If your home does not have air conditioning, your health department will tell you where you can find an air-conditioned "cooling center" where you can spend time.
- When the air quality outdoors is unsafe—during Code Orange, Code Red and Code Purple air quality alert days—limit the amount of time you spend outdoors. You can find air quality alert information online or you can download AirNow, a free app for your phone made by the EPA.
- Talk to your doctor about what else you can do to protect your health and your baby's health from the risks associated with global warming.
